# Investigation of a Negative Step Effect on Stilling Basin by Using CFD

**DOI:** 10.3390/e24111523

**Published:** 2022-10-25

**Authors:** Lei Jiang, Minjun Diao, Chuan’ai Wang

**Affiliations:** State Key Laboratory of Hydraulics and Mountain River Development and Protection, Sichuan University, Chengdu 610065, China

**Keywords:** negative-step stilling basin, hydraulic performance, 2D RANS-VOF numerical model, flow pattern, free-surface profile, velocity profile, energy dissipation efficiency

## Abstract

The negative-step stilling basin is an efficient and safe energy dissipator for high-head, large-unit discharge high-dam projects. However, studies of the effects of the negative step on the hydraulic performance of a high-dam stilling basin have not been conclusive. In the present study, a 2D RANS-VOF numerical model was developed to simulate the flow field of a negative-step stilling basin. The numerical model was validated with a physical model and then used to simulate and test the performance of the negative-step stilling basin with different step heights and incident angles. The results showed that the flow pattern, the free-surface profile, the velocity profile, the characteristic lengths are strongly influenced by the step geometry. Increasing the height of the step will increase the relative flow depth and the reattachment length in the basin, but reduce the bottom velocity and the roller length. The incident angle has no significant influence on the flow pattern of the negative-step stilling basin, and increasing the incident angle of the step will reduce the bottom velocity and the reattachment length. Both the step height and the incident angle have no significant influence on the energy dissipation efficiency because of the high submergence conditions in this study.

## 1. Introduction

In recent years, more and more high-head, large-unit discharge high-dam hydropower projects have adopted hydraulic jump stilling basins. Compared with the ski-jump energy dissipator, hydraulic jump stilling basins are more adaptable to complex terrain, less likely to cause stronger flood discharge atomization, and have less impact on the environment. However, the slab at the bottom of high-dam stilling basins is easily damaged by floods. The most notable cases of slab damage are the stilling basin of Malpaso Dam, the Karnafuli Dam, and the Wu Qiang-Xi Hydroelectric Station [1].

**Figure 1:**
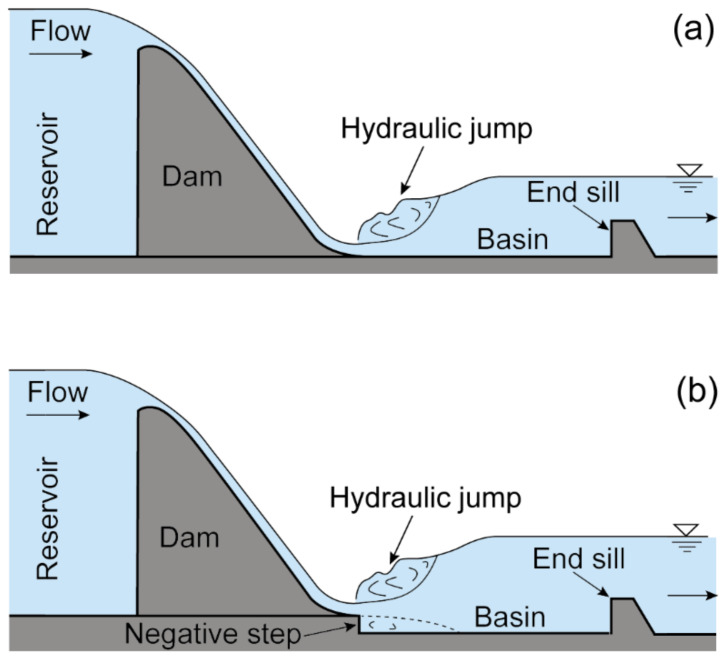
Schematic diagram of hydraulic jump stilling basins: (**a**) traditional stilling basin; (**b**) negative-step stilling basin.

Studies have shown that excessive bottom velocity is the most important cause of slab damage problems [2]. To reduce the bottom velocity, researchers have proposed applying the negative-step stilling basin (see Figure 1) to high-dam hydropower projects. Experiences of Xiangjiaba, Guandi, and other hydropower stations (as shown in Table 1) show that the negative-step stilling basin can effectively solve the slab damage problem [2,3]. However, hydraulic characteristics of hydraulic jumps at a negative step under a high-head, large-unit discharge condition have not been sufficiently investigated, which leads to difficulties for designers to accurately determine the step geometry parameters of the negative-step stilling basin of high dams.

Hydraulic jumps at a negative step and a negative-step stilling basin have been extensively studied and documented [5,6,7,8,9]. Moore and Morgan [10] showed four types of flow patterns at a negative step, that is A-jump, Wave-jump, B-jump, and minimum B-jump. Furthermore, they derived an expression for the sequent depth ratio, *Y* = *h*_2_/*h*_1_, in terms of the approaching Froude number, F_1_, and the relative pressure head on the step, *h*_d_/*h*_1_. Hager and Bretz [6] showed that the shape of the negative step did not have a significant effect on the transition of the flow regime, but the relative step height had a significant effect on the sequent depth ratios and the relative energy dissipation. The relative length of the roller can be given as a function of F_1_. Eroglu and Tastan [11] investigated the local energy losses at positive and negative steps in subcritical open channel flows. They found empirical equations for local energy losses depending on the Froude number and the relative step height. However, in the above studies, only low-head flows were considered.

In recent years, there has been an increasing number of studies on negative-step stilling basins due to the construction of many negative-step stilling basins in China. For example, Sun et al. [3] conducted a 1:90 model test with the Xiangjiaba Hydropower Station, and the results showed that the bottom velocity in the basin was significantly reduced after the use of the negative step, and the height of step had a significant impact on the bottom pressure and energy dissipation efficiency. Li [2] performed a model test study on the pressure and uplift force on the floor of the negative-step stilling basin. Jia et al. [12,13] and Huang et al. [4] investigated the pulsating pressure and wave characteristics of the negative-step stilling basin, respectively. Most of the previous research has been conducted by means of physical model tests for specific projects to analyze certain hydraulic characteristics (e.g., Bottom velocity, bottom pressure, pulsating pressure, and wave) of the negative-step stilling basin. These studies have increased the understanding of the negative-step stilling basin of high dams and successfully guided the design and operation of specific projects. However, because physical model experiments are expensive and time consuming, step geometry has rarely been used in previous studies. Therefore, there are no general conclusions about the influence of negative step geometry on the hydraulic performance of stilling basins.

Apart from the traditional experimental approach to studying hydraulic jumps, computational fluid dynamics (CFD) techniques are also useful tools with undoubtedly growing potential as computing power increases. A large number of studies on hydraulic structures in recent years have been conducted using CFD techniques. Although there are many turbulence models available, the Reynolds averaged Navier–Stokes (RANS) modeling is frequently applied to hydraulic structures including hydraulic jump stilling basins [14]. For example, Carvalho et al. [15] used RANS modeling with RNG *k-ε* closure and a two-dimensional volume-of-fluid (VOF) method to numerically model a stilling basin downstream of a spillway with a Froude number F_1_ = 6. The computed velocity, pressure obtained by this method were in good agreement with the laboratory measurements. Valero et al. [14] applied the same method to simulate the flow field of a USBR Type III hydraulic jump stilling basin under the design and adverse conditions for stepped and smooth spillways. The fine grid convergence index (GCI) as suggested by Celik et al. [16] was used in their study to perform a mesh sensitivity analysis. Zhou and Wang [17] simulated the 3D flow field among a compound stilling basin using the commercial CFD package FLOW-3D. The simulation results of four turbulence models (standard *k-ε*, RNG *k-ε*, realizable *k-ε*, and large eddy simulation turbulence models) were compared, and the RNG *k-ε* turbulence model showed the most successful agreement with the experimental results. Macian-Perez et al. [18] investigated the performance of a USBR Type II stilling basin using a CFD numerical model. The sequent depth ratio, the roller and hydraulic jump lengths, and the energy dissipation efficiency were analyzed in their study. The previous research showed that CFD complements experimental studies and the inherent measurement limitations, providing additional insight into complex flows [14]. In particular, when properly applied, RANS-based one- and two-equation turbulence models have been shown to provide accurate averaged force, distribution, and velocity fields [14]. Therefore, CFD has become an effective tool to study the hydraulic performance of stilling basins.

In general, additional research is needed regarding the influence of the negative step geometry on the hydraulic performance of the stilling basins of high dams. In this study, 2D RANS-VOF numerical simulations were performed to achieve the research goal. The initial negative-step stilling basin was designed based on the Guandi Hydropower Station. Nine negative steps with different heights (*d*) and different incident angles (*θ*) were selected to study the effects of step geometry on the flow characteristics of the negative-step stilling basin. The flow patterns, velocity profiles, characteristic lengths, and energy dissipation efficiency of the negative-step stilling basin were analyzed.

## 2. Experiment Setup

**Figure 2:**
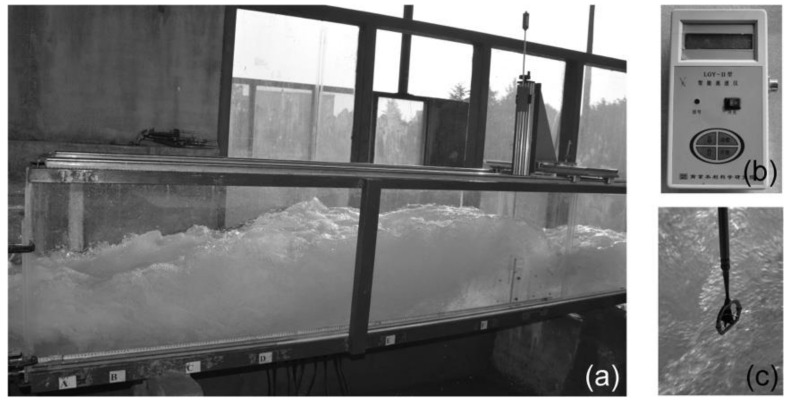
Photo of the physical model and equipment.

An initial negative-step stilling basin (Run3) was designed according to the hydraulic conditions of the Guandi Hydropower Station in the Yalong River region, Sichuan Province, China. The physical model test was carried out at a geometrical scale of 1:80. The model, consisting of a spillway and a stilling basin, was assembled in a 50-cm-wide and 60-cm-deep channel. A flap gate was installed at the downstream end of the channel to adjust the tailwater depth. The spillway and the stilling basin were made of transparent Plexiglass with a thickness of 8 mm, allowing for flow observation. The prototype spillway has gates and piers. However, in order to generalize this study, no gates or piers were installed in the model spillway, which can make the downstream flow approximately two-dimensional. Figure 2 shows the device of the physical model test.

Both the mean free-surface longitudinal profile and the maximum forward average velocity profile were measured in the physical tests. The discharge was measured by means of a calibrated V-notch weir. The mean free-surface was measured using a point gauge. The origin of the (*x*, *y*) coordinate system was located at the beginning of the stilling basin. The bottom velocity (*y* = 2.0 cm) profiles were measured using a flowmeter. The following dimensionless quantities were used for a comparison between the experiments and numerical simulations:

(1) $$X=\frac{x-x_{1}}{L_{1}}$$

(2) $$Y=\frac{y-h_{1}}{h_{t}-h_{1}}$$

(3) $$U_{b}=\frac{u_{b}}{u_{1}}$$

where $x_{1}$ is the jump toe position; $L_{1}$ is the roller length; $h_{1}$ is the flow depth at jump toe; $u_{b}$ is the bottom velocity (*y* = 2.0 cm); $u_{1}$ is the mean flow velocity at the jump toe [18].

## 3. Numerical Simulations

### 3.1. Flow Equations

The 2D Reynolds-averaged continuity and Navier–Stokes equations (RANS) were used in the present study to simulate the flow field of the negative-step stilling basins. For an incompressible, Newtonian fluid flow, flow equations can be expressed as [19]:

(4) $$\frac{\partial u_{i}}{\partial x_{i}}=0$$

(5) $$\rho \left(\frac{\partial u_{i}}{\partial t}+u_{j}\frac{\partial u_{i}}{\partial x_{j}}\right)=-\frac{\partial p}{\partial x_{i}}+\mu \frac{\partial^{2}u_{i}}{\partial x_{j}\partial x_{j}}+\frac{\partial }{\partial x_{j}}\left(-\rho \bar{u_{i}^{\prime }u_{j}^{\prime }}\right)+\rho K_{i}$$

where $u_{i}$ is the velocity component in $x_{i}$ direction; *t* is the time; ρ is the density; p is the pressure; μ is the dynamic viscosity; $-\rho \bar{u_{i}^{\prime }u_{j}^{\prime }}$ is the turbulence stresses; $u_{i}^{\prime }$ and $u_{j}^{\prime }$ are the horizontal and vertical velocity fluctuations, respectively; and $K_{i}$ is the body forces.

The turbulence stress is derived from the following linear constitutive equation [20]:

(6) $$-\rho \bar{u_{i}^{\prime }u_{j}^{\prime }}=\mu_{t}\left(\frac{\partial {\bar{u}}_{i}}{\partial x_{j}}+\frac{\partial {\bar{u}}_{j}}{\partial x_{i}}\right)-\frac{2}{3}\sigma_{ij}\rho k$$

where $\mu_{t}$ is the eddy viscosity; $\sigma_{ij}$ is the Kronecker delta; and *k* is the turbulent kinetic energy, $k=\bar{u_{i}u_{i}}/2$.

In the present numerical simulations, to determine the turbulent viscosity $\mu_{t}$ in Equation (6), the RNG *k-ε* turbulence model, based on the Boussinesq eddy-viscosity assumption, was used. The RNG *k-ε* turbulence model usually provides better results when simulating swirling flows relative to the standard *k-ε* model [21,22]. The RNG *k-ε* turbulence model expresses the turbulent viscosity in terms of the turbulent kinetic energy *k* and the turbulent kinetic energy dissipation rate *ε* as follows [23]:

(7) $$\mu_{t}=\rho C_{\mu }\frac{k^{2}}{\varepsilon }$$

where $C_{\mu }$ is the turbulence constant, $C_{\mu }=0.09$. The corresponding *k* and *ε* equations are as follows:

(8) $$\frac{\partial \left(\rho k\right)}{\partial t}+\frac{\partial \left(\rho k{\bar{u}}_{j}\right)}{\partial x_{j}}=\frac{\partial }{\partial x_{j}}\left(\Gamma_{k}\frac{\partial k}{\partial x_{j}}\right)+G_{k}-\rho \varepsilon$$

(9) $$\frac{\partial \left(\rho \varepsilon \right)}{\partial t}+\frac{\partial \left(\rho \varepsilon {\bar{u}}_{j}\right)}{\partial x_{j}}=\frac{\partial }{\partial x_{j}}\left(\Gamma_{\varepsilon }\frac{\partial \varepsilon }{\partial x_{j}}\right)+C_{1\varepsilon }\frac{\varepsilon }{k}G_{k}-C_{2\varepsilon }\rho \frac{\varepsilon^{2}}{k}-R$$

where $\Gamma_{k}$ and $\Gamma_{\varepsilon }$ are the diffusivity terms, $\Gamma_{k}=\left(\mu +\mu_{t}/\sigma_{k}\right)$, $\Gamma_{\varepsilon }=\left(\mu +\mu_{t}/\sigma_{\varepsilon }\right)$. $G_{k}$ is expressed as:

(10) $$G_{k}=-\rho \bar{u_{i}^{\prime }u_{j}^{\prime }}\frac{\partial u_{i}}{\partial x_{j}}$$

In the RNG *k-ε* turbulence model, $C_{1\varepsilon }=1.44$, $C_{2\varepsilon }=1.92$, $\sigma_{k}=1.0$, $\sigma_{\varepsilon }=1.3$, and the coefficient R in Equation (9) is given by [24]:

(11) $$R=\frac{\rho C_{\mu }\eta^{3}\left(1-\eta /\eta_{0}\right)}{1+\beta \eta^{3}}\frac{\varepsilon }{k}$$

where η is the relative strain parameters, η=Sk/ε; S is the mean strain rate, $S=\sqrt{S_{ij}S_{ij}}$; $\eta_{0}=4.38$ and β=0.012. Regarding the free surface modeling, the VOF method was adopted [25], which employs an additional variable called volume fraction (*α*) to capture the free surface at the interface between the air and the water. Based on the VOF method, *α* = 1 denotes a region that is fully inundated with water, and α = 0 corresponds to air. The free-surface volume fraction was specified to be α = 0.5 [26]. The governing equations were numerically solved using Fluent computer code (ANSYS FLUENT Academic v.19.0).

### 3.2. Numerical Simulation Runs and Solution Domain

The negative-step stilling basin numerical simulation matrix is summarized in Table 2. In Table 2, *d* is the negative step height; *θ* is the step incident angle; *Q* is the flow discharge; *q* is the unit discharge; *h*_1_, *u*_1_, and F_1_ are the mean flow depth, mean velocity, and Froude number at the toe section, respectively; and *h*_t_ is the tailwater depth. Stilling basins with six different negative step heights (*d* = 0 cm, 2.5 cm, 5 cm, 7.5 cm, 10 cm, 15 cm) and five different incident angles (*θ =* 0°, 5°, 10°, 15°, 20°) were investigated. It is important to note that the stilling basin matches the physical model dimensions. All CFD tests were performed under the design discharge *Q* = 0.09 m^3^/s (discharge per unit width is *q* = 0.18 m^2^/s), and the tailwater depth was 0.388 m.

**Figure 3:**
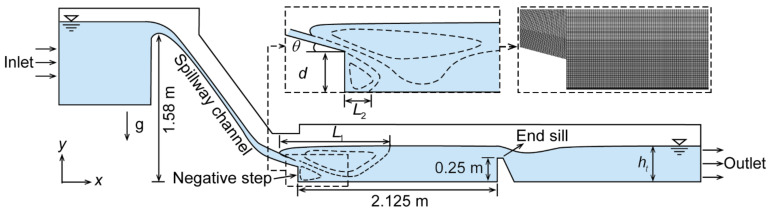
Schematic view of the calculation domain.

Figure 3 shows the schematic diagram of the calculation domain. As shown in Figure 3, the calculation domain consists of the reservoir region, spillway channel, stilling basin, and downstream region. The distance from the inlet boundary to the weir crest exceeded 10 times the height of the design heads and the outlet boundary was far enough from the end sill to ensure the formation of stable tailwater. Referring to the downstream stilling basin slab, the dam height reached 1.58 m. The length of the stilling basin was 2.125 m and an end sill of 0.25 m height was located at the end of the stilling basin. The length of the downstream region behind the height of the end sill was 2 m, which is enough to ensure that the downstream flow has sufficient distance to fully develop.

### 3.3. Numerical Settings

The explicit volume scheme was used to solve the volume fraction equation. The PISO (pressure implicit with splitting of operators) algorithm [27], coupled with a second-order backward scheme, was used to solve the momentum equation. The time term was discreted by a first-order implicit scheme [28]. A time step of Δt=0.0001 s was set for time discretization, which gave 400,000 iterations for the simulation of each case. The CFD results of each case shows that the inlet and outlet flow discharges tend to be constant when the computation time *t* > 30 s, and a total simulation time of *T* = 40 s can guarantee that each simulation is converged.

### 3.4. Meshing and Boundary Conditions

Structured mesh systems were used for the entire solution domain. For both upstream and downstream parts, a coarse mesh in the flow direction was used to reduce the number of cells and computing time [29]. Ten boundary layers contracting with a ratio of 1.1 were placed at the bottom wall of the stilling basin, and the grid point of the first layer was placed in the log law region (30<y+<200~400) [17]. A constant average velocity was used at the upstream inlet boundary for the water phase. The value of the inlet average velocity was calculated according to the design discharge (Table 2). The initial upstream water depth in the inlet boundary was given according to the test conditions. The outlet boundary was defined as a pressure outlet condition with a specified water height, which was realized through user defined functions (UDF). The upper boundary of the domain was defined as the pressure inlet condition. No-slip boundary conditions were imposed on all walls. Additionally, the standard wall function was used to compute the velocity profiles near solid boundaries [29].

### 3.5. Discretization Error Analysis

**Figure 4:**
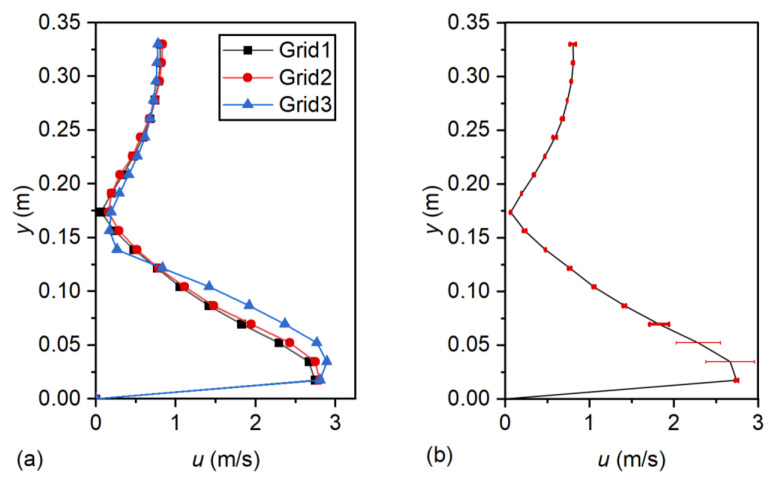
Vertical velocity profiles at *x* = 0.5 m (Run M3): (**a**) results of different meshes; and (**b**) fine-mesh solution, with discretization error bars.

Discretization error analysis was carried out based on the grid convergence index (GCI) method [16]. The GCI method can be calculated as follows [16]. Here, Run M5 was used to estimate the discretization error. Three mesh sizes, *N*_1_ = 95,900, *N*_2_ = 54,985, and *N*_3_ = 34,015, (fine mesh, medium mesh, and coarse mesh), were tested. The fine-grid convergence index can be computed as

(12) $${\mathrm{GCI}}_{21}^{\mathrm{fine}}=\frac{1.25E_{21}}{r_{21}^{P}-1}$$

where $E_{21}=\left|\left(\phi_{1}-\phi_{2}\right)/\phi_{1}\right|$ is the relative error between the medium and fine grids, where $\phi_{1}$ and $\phi_{2}$ are the fine- and medium-grid solutions for the velocity; $r_{21}=\lambda_{2}/\lambda_{1}$ is the grid refinement factor; $\lambda_{1}$ and $\lambda_{2}$ are the representative cell size of the fine- and medium-grid solutions, respectively; and *P* is the local order of accuracy. Table 3 illustrates the calculation procedure for the three selected grids. Figure 4 shows the calculation results of GCI. Computation of the GCI indicated that the discretization uncertainty in the vertical velocity profiles for the fine grid was within 11.3% (0.259 m/s), indicating good performance. Therefore, the fine-grid solution was then applied to the remaining numerical models.

## 4. Results

### 4.1. Validation of the Numerical Model

**Figure 5:**
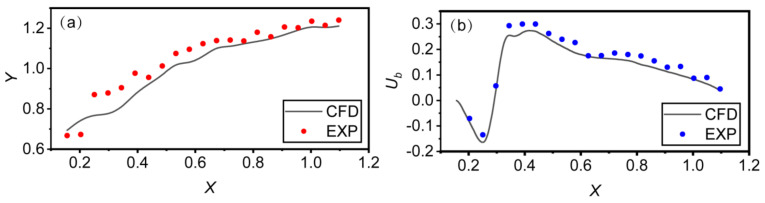
Validation of the numerical model: (**a**) mean free-surface longitudinal profile; and (**b**) bottom velocity profile (*y* = 2.0 cm).

Run M3 was selected to perform the validation of the model test and numerical simulation. The mean free-surface profile and bottom velocity profile were selected to verify the accuracy of the numerical simulations. The results of the numerical simulation and model test were compared, as shown in Figure 5. According to Figure 5, the overall trend of the simulated mean free-surface profile and bottom velocity profile agreed well with the experimental results. To quantify the reliability of the numerical simulation, the root mean square error (RMSE) and the mean absolute percent error (MAPE) were used to evaluate the accuracy of the numerical results [26]. For the mean free-surface profile, the associated RMSE and MAPE were 0.048 m and 13.2%, respectively. For the bottom velocity profile, the associated RMSE and MAPE were 0.118 m/s and 9.5%, respectively. The computed results of the statistical indicators show that the numerical simulation had high accuracy.

### 4.2. Flow Pattern

**Figure 6:**
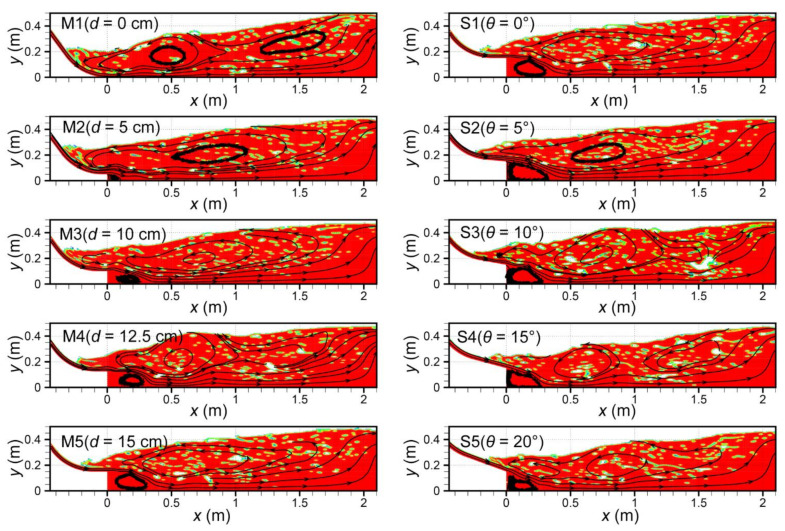
Flow pattern for the stilling basin with a negative step.

The computed flow patterns in the negative-step stilling basins are shown in Figure 6. It can be seen that due to the high tailwater depth, the toe of the hydraulic jump moved upstream into the sloping channel, and the roller lay partly in the sloping, and partly in the horizontal channel portions [30,31]. This flow pattern was defined as the B-jump [31]. For the B-jump of the negative-step stilling basin, a reattachment region is created underneath the jet, and a roller region is created above the jet. The pressure in the reattachment region is low, causing the jet to deflect to the basin bottom and eventually attach to the wall to form a wall jet. It can also be seen from Figure 6 that the area of the reattachment region increased significantly with the increase in the step height. Moreover, Run M5 had a tendency to develop toward surface jump, which indicates that the height of the can has a large influence on the flow pattern. However, the flow pattern in the basin did not change much under different incident angles.

For the B-jump, people are usually very concerned about its jump toe position. The flowing submergence parameter *E* ($E=1-y_{1}/h_{t}$), as suggested by Hager, was used to describe the jump toe location [30]. Here, $y_{1}$ is the vertical distance between the basin floor and roller leading edge; *h*_t_ is the tailwater depth. The simulation results of the submergence parameter *E* in this study are given in Table 4. It can be seen that the value of *E* was in the range of 0.29 to 0.51 and *E* did not show a regular variation with the increase in the step height and incident angle. This is mainly due to the pulsating position of the jump toe, which has a great influence on the measurement accuracy.

**Figure 7:**
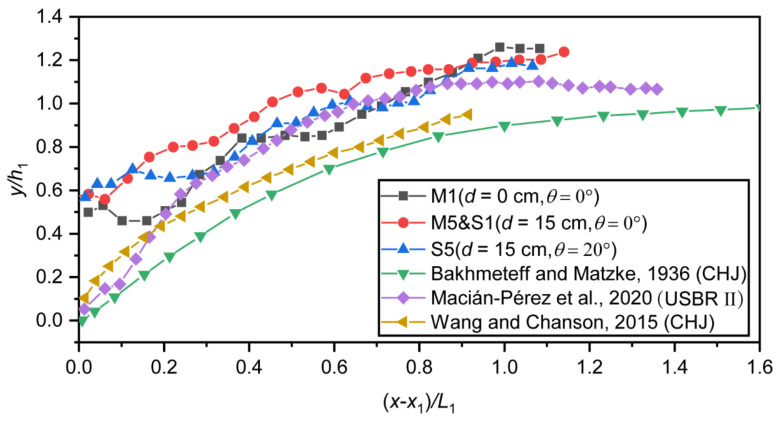
Dimensionless free-surface profile.

The results of the dimensionless free-surface profile are shown in Figure 7. The numerical simulation results were compared with the bibliographic data from three different sources. It should be noted that the profiles of Bakhmeteff and Matzke [32] and Wang and Chanson [33] were originally obtained from research on CHJ. The profiles of Macián-Pérez [34] were obtained from the USBR II stilling basin. From the figure, it can be seen that the negative-step stilling basin had a larger flow depth compared to the CHJ and USBR II stilling basin. Comparing Run M1 and Run M5, it can be seen that the flow depth in the negative-step stilling basin will increase with the height of the step, and the location of the largest increase in the flow depth is at the beginning of the basin. Comparing Run S1 and Run S5, it can be seen that the flow depth in the negative-step stilling basin will decrease with the increase in the incident angle.

### 4.3. Velocity Profile

**Figure 8:**
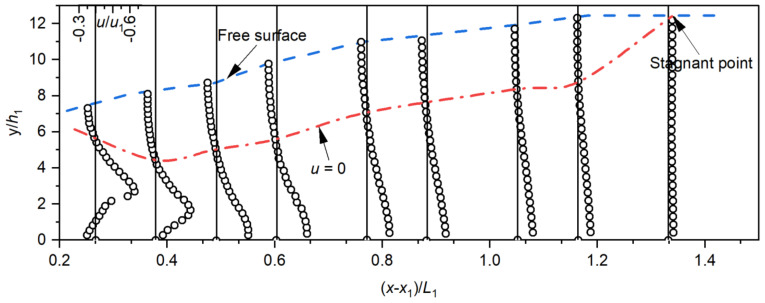
Typical vertical velocity profiles of the stilling basin with a negative step (Run M3).

The velocity profile distributions reflect the structure of the flow in the stilling basin. In this section, the velocity profiles were obtained and averaged. The influence of the step height and incident angle on the velocity profiles was analyzed. Typical vertical velocity profiles of the negative-step stilling basin are shown in Figure 8 (Run M3). The results show that the vertical velocity profiles clearly reflect the structure of the B-jump in the negative-step stilling basin. The effect of the step on vertical flow velocity is concentrated in the reattachment region (X<0.4 for Run M3). Within this range, the direction of the vertical velocity changes twice. A maximum forward velocity and a maximum backward velocity could be found at the jet core and the reverse flow region, respectively. Further downstream, the vertical velocity distribution law was gradually consistent with the typical submerged hydraulic jump vertical velocity profiles in the roller region, and a stable wall jet formed. At the end of the stilling basin, the vertical velocity distribution was similar to that of the open channel flow, with the maximum forward velocity appearing near the free surface.

**Figure 9:**
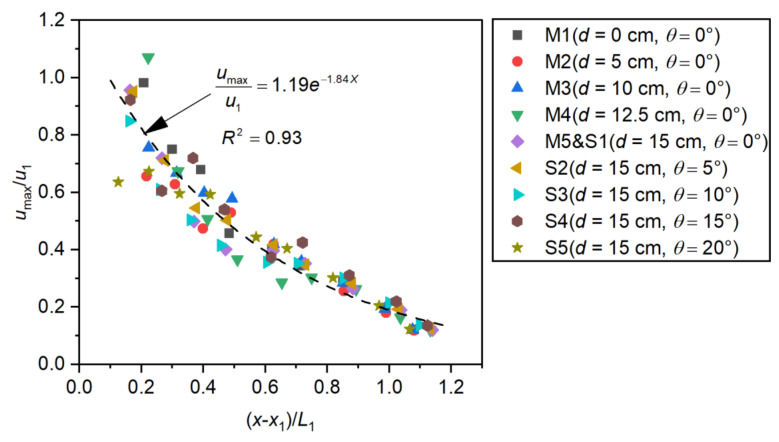
Maximum forward velocity decay along the *x*-axis direction.

The high-speed flow decreased rapidly after entering the stilling basin. The evolution of the section’s maximum velocity reflects the decay process of the flow energy, and so represents a potential cause of cavitation damage [14]. Therefore, researchers tend to use the maximum velocity as an important indicator to describe the performance of a stilling basin. In the studied cases, the dimensionless maximum velocity decay is presented in Figure 9. Here, $u_{\max}$ corresponds to the maximum velocity located at a transversal section at a distance $\left(x-x_{1}\right)/L_{1}$. From Figure 9, we can see that although the step parameters varied from case to case, the maximum velocity basically decayed along the $u_{\max}/u_{1}=1.19e^{-1.84\left(x-x_{1}\right)/L_{1}}$ ($R^{2}=0.93$) curve, and sudden changes in the maximum velocity decay were observed downstream of the negative step.

**Figure 10:**
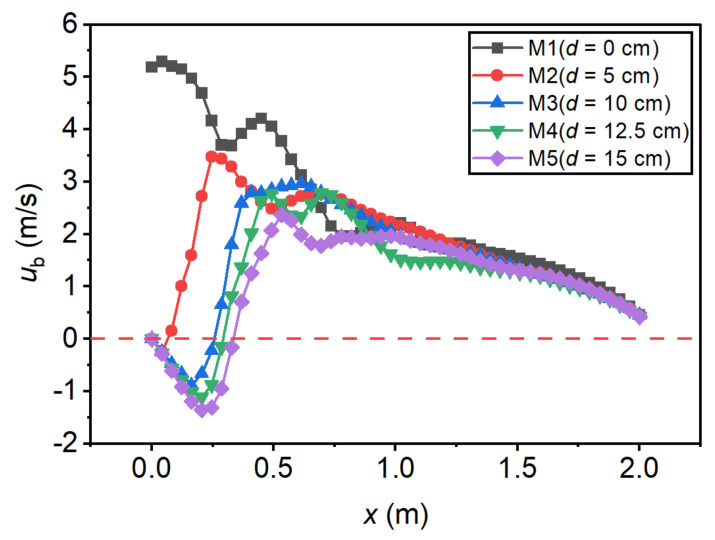
Bottom velocity profiles under different step heights.

**Figure 11:**
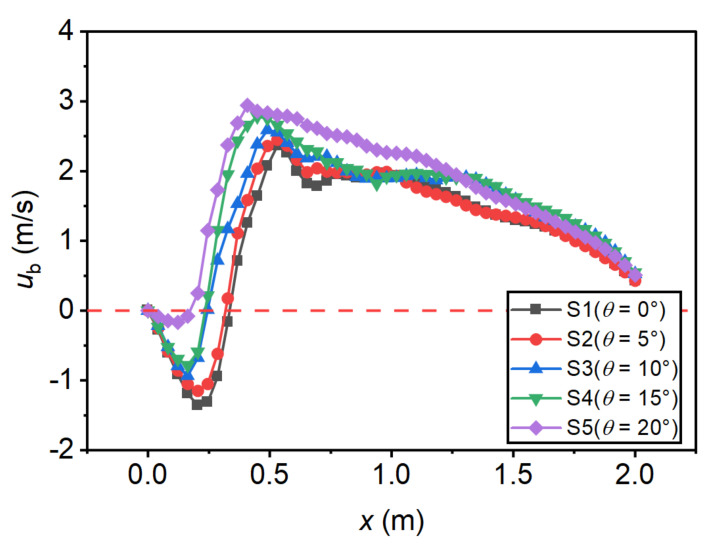
Bottom velocity profiles under different incident angles.

Bottom velocity ($u_{b}$) is also an important reference indicator for the design of the stilling basin. In the present study, the velocity at the position of y=2 cm above the basin floor was taken as the critical bottom velocity. The effect of the step height on the bottom velocity distribution is shown in Figure 10. For traditional stilling basin (Run M1), $u_{b}$ decreases along the *x*-axis direction, and the maximum bottom velocity ($u_{\mathrm{bmax}}$) is generally found at the beginning of the basin. For the negative-step stilling basin (Run M2~M5), the bottom velocity profiles were characterized by a reverse flow range (reattachment region) downstream of the basin head, and a minimum bottom velocity ($u_{\mathrm{bmin}}$) could be found in this reverse flow range. Subsequently, $u_{b}$ increases rapidly along the flow direction to $u_{\mathrm{bmax}}$. As the step height increases, $u_{\mathrm{bmax}}$ decreases gradually, and the location of the maximum bottom velocity ($x_{\mathrm{bmax}}$) moves downstream gradually. The effect of the incident angle on the bottom velocity distribution is shown in Figure 11. It can be seen that with the increase in the incident angle, $u_{\mathrm{bmax}}$ gradually increases and its position moves upstream. Table 5 lists the extreme bottom velocity and positions of each case. Comparing M1 and M5, it can be seen that $u_{\mathrm{bmax}}$ decreased by 55.3% when $d/h_{1}=0$ increased from 0 to 4.12; comparing S1 and S5, it can be seen that $u_{\mathrm{bmax}}$ increased by 24.5% when θ increased from 0 to 20°.

### 4.4. Roller Length and Reattachment Length

**Figure 12:**
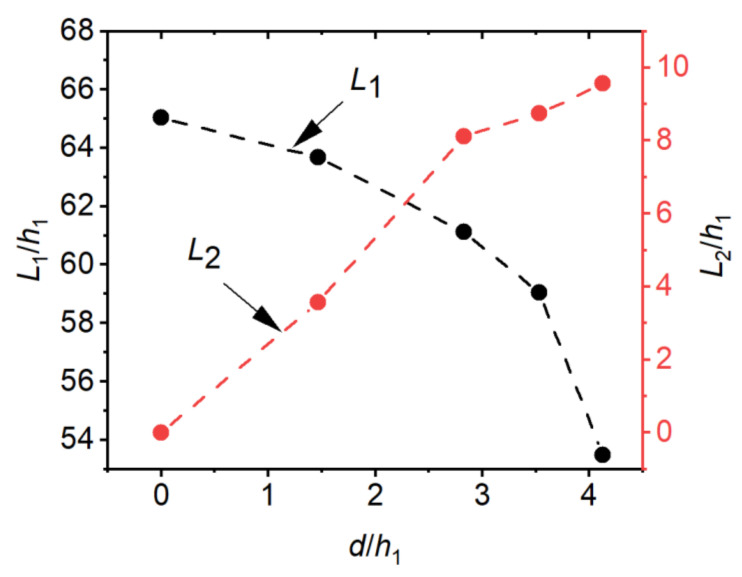
Effects of step height on the characteristic length.

The length characteristics of the hydraulic jump are usually described by the length of the surface roller or the length of the hydraulic jump. However, since the latter is not clearly defined and it is not possible to assess the length of sloping hydraulic jumps [31], the results of the length of the surface roller ($L_{1}$) are presented herein. The reattachment length ($L_{2}$) was defined as the flow distance from the source of the jet (x=0) to the point of the jet and the bottom of the basin. According to [35], the mean streamlines method was used for the reattachment length. This method defines the reattachment point as the point where the mean streamline intersects with the bottom of the basin. The effects of step height on $L_{1}$ and $L_{2}$ are shown in Figure 12. From Figure 12, it can be seen that the effect of step height on $L_{1}$ and $L_{2}$ was significant. When $d/h_{1}$ increases, $L_{1}/h_{1}$ decreases gradually, while $L_{2}/h_{1}$ increases gradually. According to the computational results, the following regression equations can be obtained to estimate the roller length and reattachment length when $d/h_{1}=0\sim 4.12$, $F_{1}=8.3\sim 9.3$, $\theta =0^{{}^{\circ}}$:

(13) $$\frac{L_{1}}{h_{1}}=-0.90{\left(\frac{d}{h_{1}}\right)}^{2}+1.20\frac{d}{h_{1}}+64.70$$

(14) $$\frac{L_{2}}{h_{1}}=2.42\frac{d}{h_{1}}+0.21$$

The predictive capability of these equations is high ($R^{2}=0.98$ and $R^{2}=0.96$, respectively).

**Figure 13:**
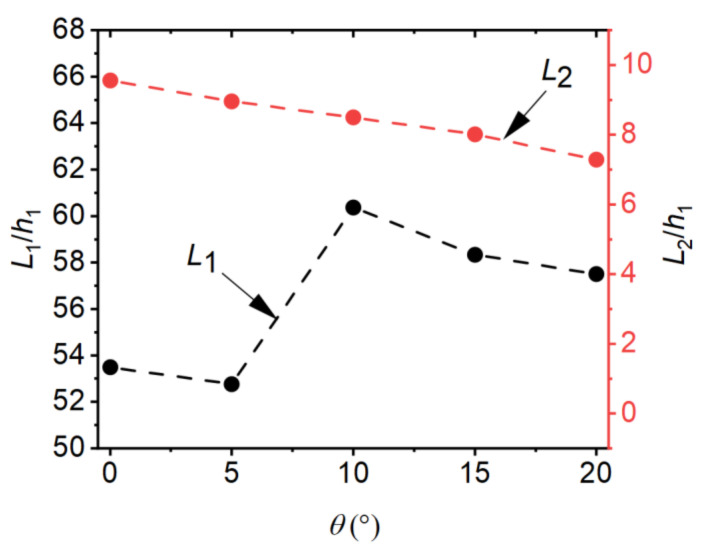
Effects of the incident angle on the characteristic length.

The effects of the incident angle on $L_{1}$ and $L_{2}$ are shown in Figure 13. From Figure 13, it can be seen that $d/h_{1}$ did not change regularly as θ increased, while it showed a linear decreasing trend. When θ increased from 0 to $20^{{}^{\circ}}$ $L_{1}/h_{1}$ only increased by 7.49%, but $L_{2}/h_{1}$ decreased by 23.7%, which indicates that the incident angle had no significant effect on the roller length, but it had a significant effect on the length of reattachment. Finally, according to the numerical calculation results, it is known that in this study, the ratio of the reattachment length to the surface roller length ($L_{2}/L_{1}$) was in the range of 0~0.18.

### 4.5. Energy Dissipation Efficiency

In this study, the energy dissipation efficiency (η) was computed in accordance with [36] as the difference between energy heads at the upstream section *x* = −2.0 m ($E_{0}$) and the downstream section *x* = 2.1 m ($E_{t}$), normalized to η

(15) $$\eta =\frac{E_{0}-E_{t}}{E_{0}}$$

**Figure 14:**
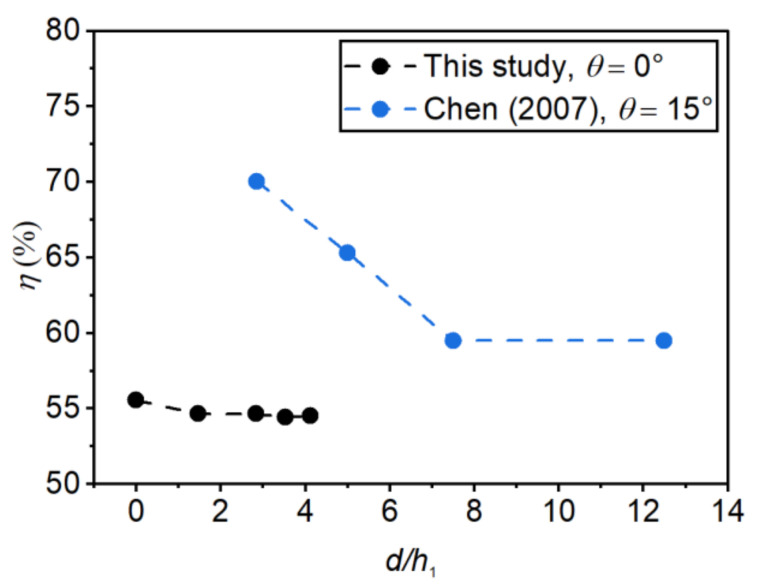
Effect of the step height on the energy dissipation rate.

**Figure 15:**
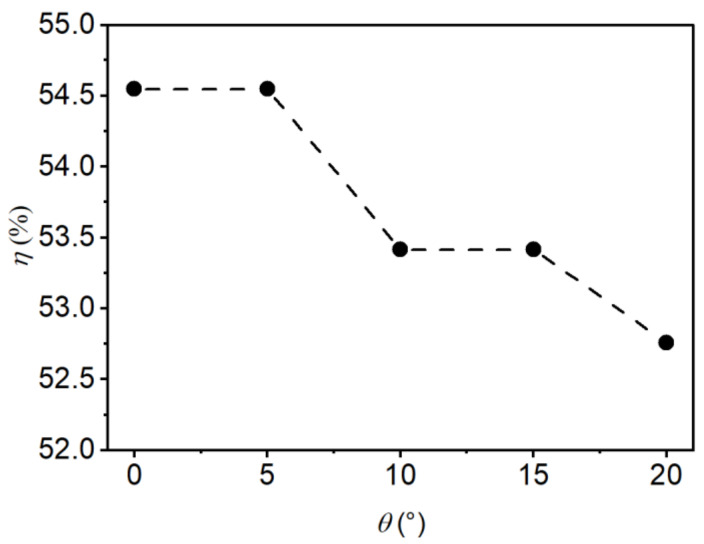
Effect of the incident angle on the energy dissipation rate.

Figure 14 and Figure 15 show η as a function of the relative step height ($d/h_{1}$) and the incident angle (θ), respectively. As Figure 14 shows, the energy dissipation efficiency decreased with the relative step height, a finding consistent with Chen’s [37] experiment. η decreased by 1.0% when $d/h_{1}$ increased from 0 to 4.12. As Figure 15 shows, the energy dissipation efficiency decreased as the incident angle increased, and the energy dissipation efficiency decreased by 1.8% as θ increased from 0 to $20^{{}^{\circ}}$.

## 5. Discussion

The present study provides a detailed description of the flow behavior of the negative-step stilling basin. The B-jump in the negative-step stilling basin is significantly influenced by the step height, while it is relatively unaffected by the incident angle. At a certain tailwater depth, a B-jump in the negative-step stilling basin will change to a surface jump when the step height increases to a critical step height ($d_{c}$) [38]. Zheng [39] proposed an empirical model of $d_{c}$:

(16) $$d_{c}=\left(4.05\sqrt[{3}]{F_{1}^{2}}-\frac{h_{2}}{h_{1}}\right)$$

A reliable design for stilling basins must consider the hydraulic jump developed within the structure [18]. Thus, surface jumps should be avoided as much as possible. That is, when designing a negative-step stilling basin, one should ensure that d is less than $d_{c}$.

The present study showed that the relative flow depths ($d/h_{1}$) of the negative-step stilling basin were greater at the same location compared to the CHJ and USBR II stilling basins. This is due to the fact that this study had a larger unit discharge and tailwater depth compared with previous studies, which resulted in a larger flow depth in the basin. In general, since the water depth in the negative-step stilling basin is higher compared to the traditional stilling basins, higher sidewalls are required to ensure the safety of the project.

The present study shows that the velocity distribution downstream of the negative step is very complex. The results of the vertical velocity distribution show that a maximum forward velocity and a maximum backward velocity can be found downstream of the step. The backward velocity is generated by the backflow formed downstream of the step. The protection of the basin floor should be increased since previous studies have indicated that there is a significant velocity gradient in the backflow area, which is also the area with the greatest pulsing pressure [2,40]. The bottom velocity was also analyzed in this study. The bottom velocity is an important indicator for the design of the basin. In China, the bottom flow velocity is required to be less than 35 m/s. The results of this study confirm that the bottom velocity can be reduced significantly by increasing the step height, and increased significantly by increasing the incident angle. This is mainly because increasing the step height will increase the jet’s movement distance in the water, greatly reducing the flow velocity of the main jet as it reaches the bottom. In contrast, by increasing the incident angle, the distance between the jet and the bottom can be shortened and the high velocity of the jet to the bottom can be maintained. This shows that the design of the negative-step stilling basin needs to consider the influence of the step height and the incident angle to achieve the most ideal design geometry.

The length of the roll reflects the concern about energy dissipation efficiency because rolling motion and associated turbulent shearing are the predominant energy dissipation mechanisms in hydraulic jumps. It may be further linked to the erosion length at the bottom, and thus to the protection range of the stilling basin. For an efficient stilling basin operation, it is advisable to use a longer roller length (thus having a larger range of high energy dissipation) [41]. According to the present findings, this may be achieved by adopting a higher step. The reattachment length is also an important characteristic length of the negative-step stilling basin, because it relates to the development of the flow downstream of the step and the size of the reattachment region [35]. The results of this study show that the reattachment length maintains a good linear relationship with both the step height and incident angle. This is important for predicting the reattachment length. However, due to the limited sample in this study, it failed to provide a good prediction equation.

The results show that the step height and the incident angle have little effect on the energy dissipation efficiency. This is mainly due to the high flow depth in the basin, which leads to a large proportion of potential energy and a small proportion of kinetic energy. Therefore, under the same upstream and downstream flow conditions, the calculated energy dissipation efficiency does not have much difference. This conclusion is more consistent with Hager’s [5] study, which concluded that when $F_{1}>8$, the relative step height no longer has a significant effect on the energy dissipation efficiency of the negative-step hydraulic jumps. In contrast, Chen’s [37] study showed that increasing the step height could significantly reduce the dissipation efficiency. This is mainly because the unit discharge and tailwater depth in Chen’s study were relatively smaller than in this study, so the energy dissipation effect was more significantly affected by the step height.

## 6. Conclusions

In the present study, the influence of a negative step on the hydraulic performance of the stilling basin of high dams was investigated for the design conditions. Nine CFD models were conducted using 2D RANS equations with a VOF model for the free-surface tracking and the RNG *k-ε* turbulence model. The results show that:

(1) The influence of step height on the flow regime is significant and the influence of incident angle on the flow regime is insignificant. Increasing the step height will lead to an increase in the flow depth in the basin, so the negative-step stilling basin needs a higher sidewall than the traditional stilling basin. However, the step height should be less than the critical height to avoid the surface jump in the basin.
(2) Increasing the step height will significantly reduce the bottom flow velocity, and increasing the incident angle will significantly increase the critical bottom flow velocity. Therefore, the design of the negative-step stilling basin needs to consider the influence of the step height and the incident angle to achieve the most ideal design geometry.
(3) Using a higher step height can reduce the roller length of the jump in the negative-step stilling basin, thus shortening the length of the dissipation basin. The reattachment length maintains a good linear relationship with both the step height and incident angle.
(4) Within the scope of this study, changing the step height and incident angle did not have a strong effect on the total energy dissipation efficiency of the negative-step stilling basin.

## Figures and Tables

**Table 1:** Representative high-head projects in China using the negative-step stilling basin [4].

No.	Name	Dam Height (m)	Design Discharge (m^3^/s)	Step Height (m)
1	Xiangjiaba	162.0	41,200	9.0
2	Huangjinping	95.5	5650	-
3	Jin’anqiao	160.0	11,668	-
4	Guanyinyuan	159.0	16,900	7.5
5	Liyuan	155.0	11,361	15.8
6	Guandi	168.0	14,000	6.5
7	Tingzikou	110.0	34,500	8.0

**Table 2:** The simulation conditions.

Run	*d* (cm)	*θ* (°)	*Q* (m^3^/s)	*q* (m^2^/s)	*h*_1_ (m)	*u*_1_ (m/s)	F_1_	*h*_t_ (m)
M1	0	0	0.09	0.18	0.033	5.386	9.4	0.388
M2	5	0	0.09	0.18	0.034	5.262	9.1	0.388
M3	10	0	0.09	0.18	0.037	5.091	8.6	0.388
M4	12.5	0	0.09	0.18	0.035	5.087	8.6	0.388
M5 (S1)	15	0	0.09	0.18	0.036	4.945	8.3	0.388
S2	15	5	0.09	0.18	0.038	4.782	7.9	0.388
S3	15	10	0.09	0.18	0.034	5.367	9.4	0.388
S4	15	15	0.09	0.18	0.034	5.301	9.2	0.388
S5	15	20	0.09	0.18	0.035	5.126	8.7	0.388

**Table 3:** Calculations of the discretization error.

*y* (m)	$\phi_{1}$	$\phi_{2}$	$\phi_{3}$	$E_{21}$	$E_{32}$	$r_{21}$	$r_{32}$	*P*	${\mathrm{GCI}}_{21}^{\mathrm{fine}}$
0.017	2.742	2.799	2.804	0.021	0.002	1.3	1.3	9.45	0.7
0.035	2.667	2.744	2.890	0.029	0.053	1.3	1.3	2.42	10.8
0.052	2.292	2.427	2.764	0.059	0.139	1.3	1.3	3.48	11.3
0.069	1.824	1.947	2.367	0.067	0.215	1.3	1.3	4.69	6.3
0.087	1.415	1.476	1.919	0.043	0.300	1.3	1.3	7.59	1.2
0.104	1.054	1.108	1.416	0.051	0.277	1.3	1.3	6.62	1.5
0.122	0.767	0.788	0.835	0.027	0.059	1.3	1.3	3.14	2.0
0.139	0.477	0.513	0.266	0.075	0.481	1.3	1.3	7.36	0.8
0.156	0.233	0.291	0.170	0.248	0.416	1.3	1.3	2.82	6.6
0.174	0.067	0.156	0.190	1.337	0.219	1.3	1.3	3.66	6.9
0.191	0.196	0.193	0.294	0.015	0.525	1.3	1.3	13.51	0.0
0.208	0.341	0.301	0.411	0.115	0.363	1.3	1.3	3.91	2.7
0.226	0.472	0.459	0.521	0.027	0.136	1.3	1.3	6.05	0.4
0.243	0.590	0.561	0.613	0.048	0.092	1.3	1.3	2.30	4.3
0.261	0.681	0.674	0.679	0.011	0.008	1.3	1.3	1.34	2.2
0.278	0.739	0.739	0.723	0.000	0.022	1.3	1.3	24.00	0.0
0.295	0.785	0.796	0.752	0.014	0.055	1.3	1.3	5.35	0.4
0.313	0.807	0.824	0.769	0.022	0.067	1.3	1.3	4.43	1.0
0.330	0.804	0.834	0.779	0.038	0.067	1.3	1.3	2.33	4.5

**Table 4:** Submergence parameters under different cases.

Run	*d* (cm)	*θ* (°)	$x_{1}\;(m)$	$y_{1}\;(m)$	*E*
M1	0	0	−0.380	0.196	0.41
M2	5	0	−0.383	0.241	0.29
M3	10	0	−0.377	0.235	0.30
M4	12.5	0	−0.365	0.229	0.32
M5 (S1)	15	0	−0.219	0.164	0.51
S2	15	5	−0.248	0.182	0.46
S3	15	10	−0.227	0.191	0.43
S4	15	15	−0.149	0.191	0.43
S5	15	20	−0.153	0.206	0.39

**Table 5:** The extreme bottom velocity and position.

Run	$x_{\mathrm{bmin}}\;(m)$	$u_{\mathrm{bmin}}\;(m/s)$	$x_{\mathrm{bmax}}\;(m)$	$u_{\mathrm{bmax}}\;(m/s)$
M1	-	-	0.041	5.293
M2	0.041	−0.239	0.245	3.473
M3	0.163	−0.892	0.612	2.954
M4	0.204	−1.110	0.694	2.784
M5	0.204	−1.356	0.531	2.365
S2	0.204	−1.148	0.531	2.448
S3	0.163	−0.932	0.490	2.588
S4	0.163	−0.781	0.449	2.785
S5	0.122	−0.169	0.408	2.944

## Data Availability

Data available within the article.

## Abbreviations

*d*	Step height
*θ*	Incident angle
*Q*	Flow discharge
*q*	Unit discharge
*h*_1_	Mean depth at the jump toe
*u*_1_	Mean velocity at the jump toe
F_1_	Froude number at the jump toe
*h*_t_	Tailwater depth
GCI	Grid convergence index
*x*_1_	Jump toe position
*L*_1_	Roller length
*h*_1_	Flow depth at jump toe
*u*_b_	Bottom velocity
*E*	Submergence parameter
*u*_max_	Maximum velocity
*L*_2_	Reattachment length
*η*	Energy dissipation efficiency
*d_c_*	Critical step height
